# Evolution of a Landscape Phage Library in a Mouse Xenograft Model of Human Breast Cancer

**DOI:** 10.3390/v11110988

**Published:** 2019-10-26

**Authors:** James W. Gillespie, Liping Yang, Laura Maria De Plano, Murray A. Stackhouse, Valery A. Petrenko

**Affiliations:** 1 Department of Pathobiology, College of Veterinary Medicine, Auburn University, Auburn, AL 36849, USA; gillejw@auburn.edu (J.W.G.); ldeplano@unime.it (L.M.D.P.); 2 Engineering Technology Research Center of Henan Province for Aquatic Animal Cultivation, College of Fisheries, Henan Normal University, Xinxiang 453007, China; 3 Department of Chemical, Biological, Pharmaceutical and Environmental Sciences, University of Messina, 98122 Messina, Italy; 4 Drug Development, Southern Research, Birmingham, AL 35205, USA; mstackhouse@southernresearch.org

**Keywords:** phage display, in vivo, landscape phage, directed molecular evolution, breast cancer, tissue-selective motifs, short linear motifs (SLiMs), elementary binding units (EBUs)

## Abstract

Peptide-displayed phage libraries are billion-clone collections of diverse chimeric bacteriophage particles, decorated by genetically fused peptides built from a random combination of natural amino acids. Studying the molecular evolution of peptide-displayed libraries in mammalian model systems, using in vivo phage display techniques, can provide invaluable knowledge about the underlying physiology of the vasculature system, allow recognition of organ- and tissue-specific networks of protein–protein interactions, and provide ligands for targeted diagnostics and therapeutics. Recently, we discovered that landscape phage libraries, a specific type of multivalent peptide phage display library, expose on their surface comprehensive collections of elementary binding units (EBUs), which can form short linear motifs (SLiMs) that interact with functional domains of physiologically relevant proteins. Because of their unique structural and functional features, landscape phages can use an alternative mechanism of directed molecular evolution, i.e., combinatorial avidity selection. These discoveries fueled our interest in revisiting the in vivo evolution of phage displayed libraries using another format of display, i.e., landscape phages. In this study, we monitored the evolution of a landscape phage library in a mouse model with and without an implanted human breast cancer tumor xenograft. As expected, the multivalent architecture of landscape phage displayed proteins provided strong tissue selectivity and resulted in a huge diversity of tissue penetrating, chimeric phage particles. We identified several types of EBU interactions that evolved during the course of tissue distribution, which included interactions of EBUs with all tissue types, those EBUs that interacted selectively with specific organs or tissues with shared gene expression profiles or functionalities, and other EBUs that interacted in a tissue-selective manner. We demonstrated that landscape phage libraries are a rich collection of unique nanobioparticles that can be used to identify functional organ and tissue-binding elements after the evolution of a phage display library in vivo.

## 1. Introduction

Soon after phage displayed peptide libraries were constructed and successfully used for “investigation of the specificity of antibodies and discovery of mimetic drug candidates” in vitro [1], they were tested in vivo, for “identifying selective endothelial markers” [2,3]. In this “adventurous” project, it was not certain whether selection of phages from phage display libraries in vivo could work similarly to in vitro experiments. Indeed, phage particles identified by in vitro methods, bearing billions of foreign fusion peptides or antibodies, interacted with immobilized targets in a stationary phase, under invariable concentrations of interacting species, and under highly controlled conditions [4,5]. On the contrary, interaction of phage-fusion peptides with epithelial cells of the vasculature in vivo occurs in a “xenophobic” environment, under vigorous blood flow (~5 cm/s) [6,7], where the chance of capturing epithelial cell receptors by a few phage fusion peptides might seem problematic. The impressive success of the maiden ”fantastic voyage” of phage libraries in a mammalian system, led to the discovery of a new family of vasculature bed-targeting peptides and suggested a novel and powerful tool for directed evolution, in vivo phage display [8,9,10]. Most in vivo phage display projects were motivated by requests for ligands, which could be used to target imaging or therapeutic nanodevices specifically to the site of disease [8,11,12,13,14]. Initially, they were focused on the discovery of ligands specifically interacting with molecular markers of organ or tumor vasculature [9,10,11,14,15,16,17,18,19]. To respond to the critical needs for construction of efficient actively-targeted delivery of anti-cancer drugs and detection probes [20], new ligands were requested that would leave the blood vasculature, either by extravasation or direct penetration through fenestrations, and interact with receptors located on the surface of cancer cells or other cell types located within the tumor microenvironment [21,22,23,24].

**Figure 1:**
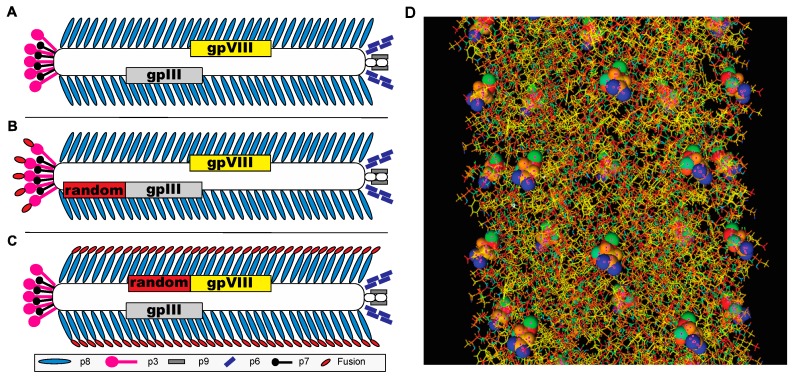
Filamentous phage display libraries. Schematic representation of type-3 and type-8 phage display libraries on the parent fd-tet bacteriophage vector. Each phage library is composed of a collection of phages with a randomized peptide fusion (red) displayed on every copy of the p3 or p8 protein. (**A**) Wildtype fd-tet vectors composed of 4000 copies of the p8 major coat protein (blue) encapsulating a single stranded DNA genome. Ends of phage particles are enclosed by 4 minor coat proteins: p3 (pink), p6 (black), p9 (grey), and p7 (purple). (**B**) Display of fusion peptides (red) on every copy of the fd-tet p3 protein, present in 5 copies per phage. (**C**) Display of fusion peptides (red) on every copy of the fd-tet p8 protein, present in ~4000 copies, generating a library of phage particles with unique peptide landscapes for interaction with the environment. (**D**) Stick model diagram of an ~10 nm segment of a type-8 landscape phage. Fusion peptide sequences are displayed at the N-terminus of each copy of the p8 protein and modeled as spheres to demonstrate the unique landscapes generated by each particle.

Multiple advanced in vivo selection and affinity maturation strategies have been used to discover peptides that bind or internalize into tumor cells of interest [21,23,25,26]. The majority of protein-based, directed evolution projects traditionally prefer the use of filamentous bacteriophages as a carrier of diverse libraries of fusion peptides or antibodies [5,27,28,29]. The tubular capsid of filamentous phages is composed predominantly of the major coat protein p8, which contributes ~90% to the virion mass, and minor coat proteins (~1% to 2% of the virion mass) at the ends of the phage shell (Figure 1A). In a phage display vector, a foreign DNA fragment is placed into one of the coat protein genes, so that the encoded peptide becomes fused to the corresponding coat protein, and thus displayed on the exterior of the capsid (Figure 1B,C). A phage display library is a population containing billions of clones, each displaying a distinct fusion peptide or antibody domain on the phage surface [5,29]. The p3 and p8 phage coat proteins of fd bacteriophage, generally used for phage display, are represented by 5 and ~2700 copies (~4000 copies in fd-tet based vector systems), respectively (Figure 1A). The p3-displayed libraries are commonly preferred for the selection of high affinity peptides or antibodies using an affinity selection procedure referred to as biopanning [4]. Alternatively, multivalent p8-fusion libraries, also called landscape phage libraries [30,31], allow selection of peptides with lower affinities but strong overall binding due to avidity that hides the lower affinities found with individual peptide ligands [32]. Because of their multivalency, landscape phage libraries demonstrate a high propensity towards combinatorial avidity selection, an alternative mechanism for directed molecular evolution, revealed during in vitro selection of MDA-MB-231 breast cancer cell-binding and penetrating phages [33].

In previous experiments of the evolution of phage display libraries in vivo, the difference in phage biodistribution to different organs and tissues was attributed mostly to the structure of the displayed peptide or antibody fusions to the p3 of filamentous phages M13 or fd, while physical and chemical properties of the vectors, as naturally-occurring nanomaterials, were mostly disregarded [19,26]. In this paper, we studied the in vivo evolution of a p8-type landscape phage library in a mouse model. Traditionally, the term “evolution”, as is used in the context of phage display affinity selection and maturation, is used to describe the multistage process of phage enrichment through multiple, discrete rounds of selection to select phages with the strongest binding to a target [5]. With our prospective goal of using phages to identify molecular programs for tissue navigation [33,34], landscape phages became a versatile research instrument because of their intrinsic plasticity in interactions with protein domains through combinations of elementary binding units (EBUs) [35] and short linear motifs (SLiMs) [36,37,38]. This unique propensity of landscape phages to be enriched in reversible, SLiM-mediated interactions within tissues and the tumor microenvironment (TME) allowed us to extend and apply the use of the term “evolution” to describe the multi-pass circulation of the library within the bloodstream as a form of in vivo evolution of the landscape phage library for tissue migration. The results of phage migration within each tissue microenvironment can be attributed to accumulation of EBUs following unforeseen steps in a directed evolution scheme for tissue-selective navigation. On the basis of the underlying mechanism of EBU enrichment, it is conceivable that some EBUs evolved through this scheme could be involved with increased solubility or improved pharmacokinetic profiles allowing better retention of phages in circulation, while others may be involved with increased extravasation from blood vessels and their penetration into the tissue microenvironment. Finally, other EBUs may mediate the binding interactions with specific cell types within each tissue or organ.

Our interest in studying the evolution of a landscape phage library in vivo was fueled by the highly structural variability of the component phage virions, determined by the dominance and high diversity of the p8-fusion phage proteins. We assumed that the chemical diversity observed within the landscape phage libraries, as revealed in our in vitro migration experiments, could also affect the specificity of tissue distribution of phage and phage-derived nanoparticles in vivo [39,40]. Therefore, our primary goal of this study was to demonstrate that peptide fusions to the filamentous bacteriophage (fd-tet) major coat protein (p8) would produce dramatic changes to the exposed surface of each phage particle and lead to phage particles with unique migration properties. In particular, we studied the ability of various filamentous phage libraries to migrate through a structurally simple microenvironment in response to an externally applied electric field in vitro and the tissue distribution of a landscape phage display library in vivo using a mouse xenograft model of the MDA-MB-231 human breast cancer cell line.

## 2. Materials and Methods

### 2.1. General Landscape Phage Procedures

The multibillion-clone, type-8 landscape phage display library f8/9 was constructed using the f8-6 vector to display a 9 amino acid peptide fusion to the N-terminus of the mature p8, present in 4000 copies, as described previously [41]. The f8/9 phage library is composed of ~1.2 × 10^9^ different phage variants, each displaying a structurally unique peptide sequence with novel and emergent properties to be discovered. Common phage methods, including phage isolation, propagation, purification, tittering, and Sanger sequencing of phage gDNA were described previously [42].

### 2.2. Agarose Gel Electrophoresis of Phage Libraries

Phage libraries or isolated phage clones (~10^10^ virions per sample) were separated on a 0.8% agarose gel in 4× GBB (pH 8.3) at a constant 20 V (~1.38 V/cm) for 24 hours at 4 °C as previously described by [43]. Electrophoresed gels were soaked in 0.2 N NaOH for 1 hour at room temperature to denature phage particles, washed with ddH_2_O for 5 minutes, and neutralized with 1 M Tris-HCl, pH 7.0 for 15 minutes. Gels were then soaked in 3× GelRed (Biotium, Fremont, CA, USA) staining solution diluted in ddH_2_O for 1 hour at room temperature and briefly rinsed with ddH_2_O for 5 minutes. Phage ssDNA bands were visualized using the UV imaging tray on a GelDoc Imaging Station (Bio-Rad, Hercules, CA, USA).

### 2.3. Animals

Athymic nude mice (NCr-nu/nu), 4 to 6 weeks old, were obtained from Charles River Laboratories (Frederick, MD, USA). All mice in this study were group housed in microisolator cages (≤5/cage) at an AAALAC-accredited animal facility (#000643) within Southern Research (SR; Birmingham, AL, USA) operating under a standard 12 h light and dark cycle. Mice had access to sterilized food pellets and water ad libitum and no additional consumable enrichment was provided. All animal experiment procedures were reviewed and approved by the Institutional Animal Care and Use Committee (IACUC) of SR (approval #15-03-009B).

After acclimation, mice were implanted subcutaneously with a small fragment of human MDA-MB-231 breast cancer tumors (30–40 mg) derived from an in vivo passage in mice into the right flank. Mice were monitored daily for mortality and moribundity, with tumor volumes being assessed twice weekly until tumor volumes were ~750 mm^3^ (range 301.2–1631.7 mg). Mice with appropriately sized tumors were randomly assigned to treatment groups (*N* = 3 per group, 12 total mice).

### 2.4. Library Distribution and Tissue Collection

A portion of the multi-billion clone phage display library f8/9 was diluted with 1× PBS to prepare two treatment groups (*N* = 3 per group) containing either a high dose (4.0 × 10^10^ virions) or medium dose (4.0 × 10^9^ virions) of phages. Mice received a single intravenous injection of phage library via lateral tail vein in a constant volume of 200 µL per injection. Mice were returned to their cages and the landscape phage library was allowed to circulate for 1 or 24 hours (*N* = 3 per group) before sacrifice and necropsy. At the determined time point, mice were deeply anesthetized and blood collected via cardiac puncture for isolation of serum. Mice were immediately perfused with at least 5 volumes of 0.9% saline solution until tissues were blanched. Tissues (brain, heart, kidneys, liver, lungs, pancreas, spleen, and tumor) were recovered, weighed, and bisected into two pieces. Half of each tissue was flash frozen in liquid nitrogen for molecular analyses, including: biological titering of infectious phages, amplification of phage sublibraries, qPCR of total phage genomes, and next-generation sequencing of phage populations enriched in each tissue. The second half of each tissue was fixed in 10% netral-buffered formalin (NBF) for 24 hours, trimmed, processed, and embedded into paraffin blocks for archiving and histological analysis.

### 2.5. Tissue Processing and Archiving

Recovered mouse tissues were processed as previously by [44]. Briefly, flash frozen tissues were thawed at room temperature and dissected into ~100 mg fragments with a sterile blade before transferring into a weighed, 1.7 mL microcentrifuge tube. Each tissue fragment was homogenized in 500 µL of ice-cold homogenization buffer (Eagle’s Minimal Essential Medium, EMEM, supplemented with 0.5% Bovine Serum Albumin, BSA) using a disposable pestle and handheld pellet mixer without generating aerosols. Dispersed tissues were centrifuged at ~6000× *g* for 5 minutes at room temperature and the supernatant discarded. Homogenized cell pellets were washed with 1 mL of ice-cold homogenization buffer (EMEM with 0.5% BSA) three times until the resulting supernatant was clear with the supernatant being discarded after each wash. Cell pellets were weighed before resuspending in 250 µL of 3-[(3-cholamidopropyl)-dimethylammonio]-1-propanesulfonate (CHAPS) lysis buffer (2.5% CHAPS in EMEM supplemented with 0.5% BSA) to extract phages from tissues. Each sample was incubated in a cold room at 4 °C for 1 hour with gentle rotation to allow complete tissue lysis and release of phage particles.

A portion of total DNA for molecular analysis and archiving was recovered from each tissue sample using DNAzol reagent (Invitrogen, Carlsbad, CA, USA) according to the manufacturer’s instructions. Briefly, 100 µL of each homogenized sample was transferred to a sterile, 1.7 mL microcentrifuge tube containing 1 mL of DNAzol reagent and thoroughly mixed. Cell debris was precipitated by centrifugation at 10,000× *g* for 10 minutes at room temperature and the cleared supernatant was transferred to a new 2.0 mL microcentrifuge tube. DNA was precipitated by addition of 500 µL of 100% ethanol, inverting 10 times, and incubation at room temperature for 3 minutes. Samples were centrifuged at ~6000× *g* for 5 minutes at room temperature to recover DNA and the supernatant was removed. Resulting DNA pellets were washed twice by gentle inversion 5 times in 800 µL of 75% ethanol and then centrifuged at ~1500× *g* for 2 minutes. Residual ethanol was removed, and the DNA pellet was resuspended in 200 µL of freshly prepared 8.0 mM NaOH solution. After the DNA was completely dissolved, the solution was neutralized to a pH of ~7.2 by addition of 4.6 µL of 1.0 M HEPES (4-(2-hydroxyethyl)-1-piperazineethanesulfonic acid) solution. The final solution was clarified of insoluble material by centrifugation at 15,000× *g* for 10 minutes at room temperature and transferred to a new microcentrifuge tube for archiving. DNA concentration and purity were assessed by UV/Vis spectroscopy.

### 2.6. Quantification of Infectious Phages in Tissues by Titering

The concentration of intact, infectious bacteriophage particles contained within each tissue was determined by titering transducing units (TU) in K91BluKan *E. coli* cells as previously, with minor modifications, by [44]. K91BluKan *E. coli* starved cells were prepared according to standard protocols, with cell infectivity determined by dividing the biological titer (TU/mL) by the physical titer obtained by UV/Vis spectroscopy (vir/mL) [42]. Homogenized tissue samples were diluted with cold homogenization buffer (EMEM + 0.5% BSA) and 10 µL of each dilution was titered in an equal volume of *E. coli* starved cells. Infection proceeded at room temperature for 15 minutes following by induction of tetracycline resistance genes by incubation with 180 µL of NZY medium with 0.2 µg/mL tetracycline for 45 minutes at 37 °C. Cell suspensions were then plated on selective NZY/Kan/Tet agar plates. The number of TU were counted the following morning to calculate the biological titer (TU/mL). The number of TU per amount of tissue (TU/g) was calculated by dividing the number of TU in 10 µL by the mass of tissue (g) present in 10 µL of homogenized sample. To normalize variability of infectivity measurements between multiple experiments, the number of TU were converted to a corrected physical number of particles (vir) by dividing the number of TU by the infectivity obtained in that experimental batch of cells with a standardized concentration of fd-tet phage.

### 2.7. Quantification of Total Phage Particles in Tissues by qPCR

Concentrations of recombinant phage derivatives based on fd-tet phage (NCBI GeneBank AF21317.1) were measured by quantitative SYBR Green I assay using gp8-specific primers and following MIQE guidelines [45]. Briefly, DNA oligonucleotides used for amplification of gp8-specific amplicons, f8s-20 forward primer [5′-CAAAGCCTCCGTAGCCGTTG-3′], and f8as 20 160 reverse primer [5′-AATGACAACAACCATCGCCC-3′] were synthesized and purified using standard desalting methods by Integrated DNA Technologies (IDT), and resuspended at 10 pmol/µL in ddH_2_O. Twenty microliter reactions were manually prepared for each sample in white 0.2 µL PCR tubes with optically clear lids (USA Scientific TempAssure #1402 4780) and were composed of Quanta PerfeCTa SYBR Green FastMix (1×), f8s-20 forward primer (250 nM), f8as-20-160 reverse primer (250 nM), and 2 µL of diluted template. Serial dilutions of control phage (fd-tet), positive control (propagated recombinant phage clone of known concentration), negative control (K91BlueKan *E. coli* gDNA), and no template control (NTC) samples were prepared in duplicate and each unknown sample was prepared in triplicate for each assay. Samples containing phage particles were diluted with ddH_2_O as needed. Serum samples were diluted 1:100, total DNA extracted from tissue samples were diluted to <50 ng/µL, sublibraries enriched following in vitro selections against MDA-MB-231 cells were diluted 1:100 [33], and a portion of primary f8/9 phage library was diluted 1:1000. Positive control and negative control samples were diluted 1:100 with ddH_2_O. Ten-fold serial dilutions of known concentrations of fd-tet phage were prepared in ddH_2_O in the range of ~1.0 to ~1.0 × 10^7^ virions/µL. As an internal control for all qPCRs and to normalize for variations in template concentration between samples, the concentration of mammalian gDNA was measured in a separate reaction using UC.33-specific primers using the same reaction composition. DNA oligonucleotides used for amplification of UC.33-specific amplicons, UC.33 1F forward primer [5′-AACTTGCCCCAATTAACCGC-3′], and UC.33 1R reverse primer [5′-AGACTTTGGGGCGTAACCAT-3′] were prepared, as above. Ten-fold serial dilutions of gDNA isolated from human MCF-7 cells in the range 0.005 to 50 ng/µL were also prepared. All qPCRs were conducted on a C1000 Touch CFX 96 real-time detection system (Bio-Rad) using a standard two-step amplification profile followed by a melt curve analysis as follows: initial melting at 95 °C for 3 min, 40 cycles of 95 °C for 10 s and 60 °C for 30 s, a final elongation step at 72 °C for 5 min, and finally a melt curve analysis from 75 °C to 95 °C in 0.2 °C increments for 10 s each. Reaction specificity was verified by melt curve analysis and agarose gel electrophoresis of at least one replicate for each sample. Quantification cycle (C_q_) threshold was determined by automated calculation (Bio-Rad CFX Manager; v. 3.0) and verified visually to be higher than the baseline background signal and also within the linear amplification range for each reaction. A standard curve was prepared using 8 serial dilutions of fd-tet phage or 5 serial dilutions of MCF-7 gDNA. Unknown recombinant phage concentrations were determined from a linear regression model obtained from the standard curve and reported as the mean concentration (vir/µL) ± sample standard deviation. Similarly, unknown mammalian gDNA (mgDNA) concentrations were determined and reported as the mean concentration (ng mgDNA/µL) ± sample standard deviation. Number of phage genomes per mass of tissue (vir/g) were calculated by dividing the phage concentration by the concentration of mgDNA and multiplying by the amount of gDNA recovered from a given mass of tissue (ng mgDNA/g tissue). Injected dose (ID/g) was calculated by dividing the amount of phage per mass of tissue (vir/g) by the injected dose (vir).

### 2.8. Next-Generation Sequencing of Phage Populations Enriched in Tissues

A portion of phage gp8-specific amplicons isolated from each tissue were purified by QIAquick PCR purification kit (Qiagen, Hilden, Germany) according to the manufacturer’s specifications and eluted with nuclease-free ddH_2_O. Purified gp8-specific amplicons were shipped to the Massachusetts General Hospital Next Generation Sequencing (MGH NGS) core facility (Cambridge, MA, USA) for construction of sequencing libraries, quality control checks, and massively parallel sequencing. Unique barcodes and adapters for Illumina NGS platforms were added to each sample using the NEBNext Multiplex Oligos for Illumina kit (New England Biolabs, Ipswich, MA, USA). Final amplicon libraries with multiplex adapters to identify different phage populations within each tissue, were analyzed for purity, fragment size, and concentration using a high sensitivity D1000 ScreenTape run on a Tapestation 4200 (Agilent, Santa Clara, CA, USA). Ten multiplexed, gp8-specific amplicon libraries were pooled into a single lane with a 15% Phi-X control library to increase the low sequence diversity expected at the initiation of the sequencing run. Pooled samples were sequenced via 150 bp, single-end (SE) sequencing run on a MiSeq (Illumina, San Diego, CA, USA) instrument using a 150 bp MiSeq Reagent Kit v3 (Illumina). Raw, demultiplexed FASTQ files were obtained from MGH via file transfer protocol (FTP) server for bioinformatics analysis. Raw single-end sequence data in FASTQ format was deposited and made publicly available in the National Center for Biotechnology Information’s (NCBI) Short Read Archive (SRA) database with links available from the NCBI BioProject database via accession number PRJNA576688.

**Figure 2:**
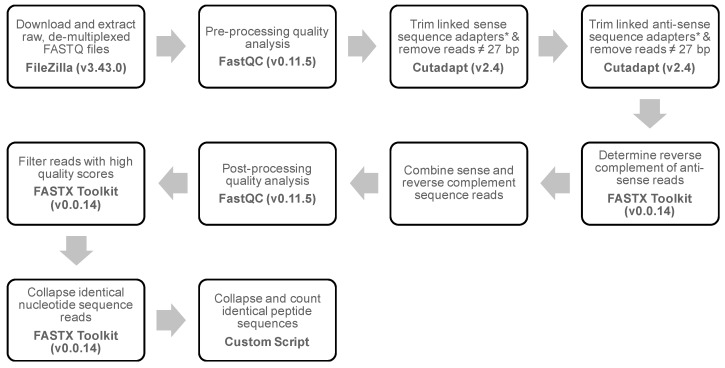
gp8-Targeted next-generation sequencing bioinformatics workflow. Schematic for identification of displayed peptide sequences from landscape (type-8) phage libraries via next-generation sequencing. Briefly, raw nucleotide sequence reads are trimmed for linked sense/anti-sense upstream and downstream regions of gp8 and filtered based on the expected nucleotide insert size. High quality sequence reads are collected and collapsed into a representative peptide sequence with metadata indicating number of nucleotide variants encoding each peptide.

A gp8-targeted NGS bioinformatics workflow was developed to isolate and analyze the nucleotide fusion sequences introduced into p8-type phage display libraries, as summarized in Figure 2. Overall performance of the sequencing run was assessed using FastQC (v. 0.11.5) to verify read length and sample quality scores [46]. Sense reads were trimmed for linked gp8 upstream and downstream sequences from f8/9 library-derived phages [5′-ATGCTGTCTTTCGCTGCAN_27_CCCGCAAAAGCGGCCTTTGACTCCCTGCAAGCTAGCGC-3′] and filtered for trimmed reads with a 27 bp oligonucleotide size using CutAdapt (v. 2.4) [47]. Similarly, anti-sense reads were recovered, trimmed for linked anti-sense gp8 upstream and downstream sequences [5′-GCGCTAGCTTGCAGGGAGTCAAAGGCCGCTTTTGCGGGN_27_TGCAGCGAAAGACAGCTA-3′] and filtered for size as above. The Fastx Reverse Complement program of the FASTX-Toolkit (v 0.0.14) was used to find the reverse complement of each anti-sense read [48]. Sense and reverse complement anti-sense reads were combined into a single file of sense reads and post-processing quality was assessed using FastQC as above. Reads with a low-quality score (Q < 34) were removed using the Fastq Quality Filter program and identical reads were collapsed into a single representative sequence using the Fastx Collapser program [48]. Peptide sequences were determined by translation of the primary nucleotide sequence and then duplicates collapsed into a single peptide record with a custom Python 3 script using functions from the BioPython (v. 1.74) library [49,50].

### 2.9. Bioinformatics Analysis of Tissue-Selective Phages

Motif frequencies were revealed using a custom Python 3 script to count the number of unique, overlapping 3-mer amino acid sequences that were derived from each tissue sample and reported as the percentage of total motifs recovered from each tissue sample. A hierarchically clustered heatmap was generated as a visual representation to compare motif frequency between all tissue samples. The clustermap function from the seaborn library (v. 0.9.0) was used to arrange motifs into a heatmap, grouping motifs with similar occurrence rates, and clustering tissues with similar profiles with Ward’s clustering method [51]. Motifs with low tissue frequencies, less than 0.0125% (1/8000) in all samples, were removed from the final visualization.

To identify tissue-selective 3-mer motifs observed in each tissue, we performed a Fisher’s exact test on each motif, as suggested previously by [52]. For each identified motif recovered per tissue, a 2 × 2 contingency table comparing the frequency of each motif observed in the isolated tissue with the frequency of each motif observed in the normalizing sample was prepared and a *p*-value calculated using a one-sided Fisher’s exact test, implemented in a custom script using the Fisher exact function from the scipy (v. 1.3.1) library [53]. We compared the performance of three normalization schemes to identify tissue-selective motifs, including normalization with (1) unselected primary f8/9 library, (2) all other tissues, and (3) serum (Figure S2). *P*-values were corrected for multiple comparisons using the Benjamini–Hochberg method to control the false discovery rate [54]. Motifs were plotted in a one-sided volcano plot and considered tissue-selective if the adjusted *p*-value was <0.05 and motif enrichment in the tissue was >2-fold as compared with all other tissues.

To identify interaction networks between EBUs within the same peptide, we used network analysis of significantly enriched tissue-selective 3-mers as nodes in an undirected network. EBU nodes were connected by weighted edges representing the frequency of unique peptides containing both 3-mers in the same structure. The network was generated using a custom Python script using the NetworkX (v. 2.3) library and visualized using Gephi (v. 0.9.2). Edges with weights less than 20 were filtered from the visualization for improved display of more significant network interactions. All scripts were deposited in a publicly available GitHub software repository (https://github.com/gillejw/PDBT; doi:10.5281/zenodo.3478383).

## 3. Results

### 3.1. Migration of Phage Display Libraries through a Simple Media

**Figure 3:**
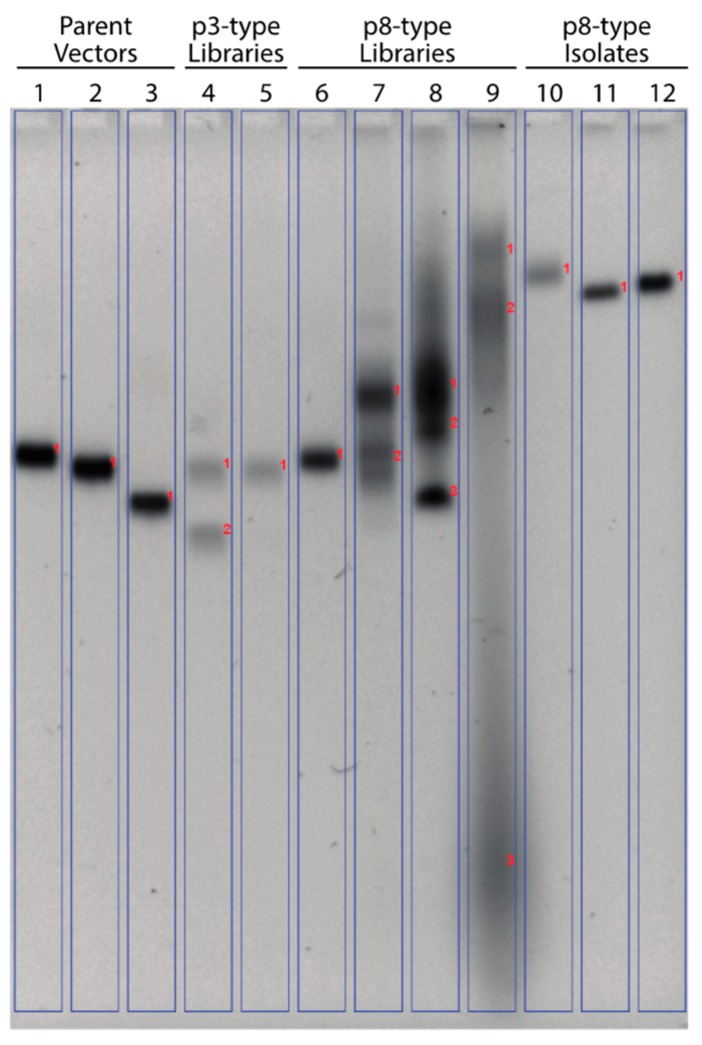
Migration of intact phages in gel electrophoresis. Relative mobility of intact filamentous phage display libraries and isolated phage clones by electrophoresis through an agarose gel. Parent phage vectors: fd-tet (lane 1), f8-5 (lane 2), or fd-cat (lane 3); p3-type libraries displaying a 6-mer (f3-6mer, lane 4) or 15-mer (f3-15mer, lane 5); p88-type library displaying a 15-mer fusion on ~10% of p8 proteins (f88-15mer, lane 6); p8-type libraries displaying a 6-mer (f8-5/6mer, lane 7), 8-mer (f8-4/8mer, lane 8), or 9-mer (f8/9, lane 9); and isolated clones from the f8/9 library displaying the fusion peptides DMPGTVLP (lane 10), EPSQSWSM (lane 11), or VPEGAFSS (lane 12). Bands are marked with red numbers to aid in discussion.

Isolated landscape phages displaying an N-terminal peptide fusion to the p8 major coat protein, present in 4000 copies, demonstrated a significant change in their mobility relative to the intact phage particles and phages which display a peptide fusion on the minor coat protein p3 through an agarose gel. This effect can be attributed to dramatic global changes of the landscape phage surface charge due to ionization of thousands of different amino acid residues exposed to the local environment [43]. To compare how this effect can influence mobility of dispersed populations of phage displayed peptide libraries of p3- and p8-types, samples containing ~10^10^ intact bacteriophages from three different parent vectors, six phage display libraries and three isolated phage clones from the f8/9 landscape library were electrophoresed as intact phage particles through a 0.8% agarose gel, as shown in Figure 3.

First, we compared the migration of two commonly used vectors used to construct phage display libraries, fd-tet phage (Figure 3, lane 1) and f8-5 (Figure 3, lane 2). Phages generated from these parent vectors produce particles of identically sized genomes (Table S1) but differ in amino acid sequence of the mature major coat protein by a single mutation (D4E) visible in f8-5 phages, which causes the surface of f8-5 phages to be more negatively charged. Following electrophoresis, f8-5 phages migrated slightly faster towards the anode than the wildtype fd-tet phages demonstrating the effect of a single amino acid substitution to the exposed regions of p8, amplified across 4000 residues per phage, to modify the physicochemical properties of the phage particle. Next, we compared the effects of peptide fusion length in p3-type libraries on migration. Phages displaying a 6-mer (Figure 3, lane 4) or a 12-mer (Figure 3, lane 5) peptide fusion to p3 have slightly different genome sizes but express the same wildtype p8 giving identical surface profiles to each of the phage particles in the respective libraries. After electrophoresis, the upper band produced by each library (band 1) migrate to the same position, demonstrating that addition of a fusion peptide, of any length, to p3 does not significantly change the physicochemical properties of the phage particle. A second band (Figure 3, lane 4, band 2) with higher mobility was identified in the f3-6mer library and is thought to be smaller phage particles as a result of phage genome instability and the possible loss of the Tn10 gene cassette.

As modification of p3 produced no significant changes to the physicochemical properties of the phage particle, we next studied the effects of p8 modification on relative mobility. Phage derived from the f88-15mer phage library (Figure 3, lane 6), containing a 15-mer peptide fused on ~10% of p8 proteins, represent particles with a low level of p8 modification and demonstrated no significant change in mobility as compared with the particles of the f8-5 vector phage (Figure 3, lane 2). Phage libraries containing particles with completely modified p8 were tested, comparing the effects of peptide fusion length on migration. As the length of the fusion protein was increased from a 6-mer (Figure 3, lane 7) to a 9-mer (Figure 3, lane 9), a significant change in mobility was observed from a single diffuse band with delayed relative mobility (Figure 3, lane 7, band 1) to multiple largely diffused bands with both delayed (Figure 3, lane 9, bands 1 and 2) and increased relative mobility (Figure 3, lane 9, band 3). Particles observed from the f8/9 library with relatively higher mobility may also belong to a satellite library of microphages with a reduced genome size than other members of the library [55,56]. It was also possible to distinguish vector-containing clones in two of the p8 libraries, f8-5/6mer (Figure 3, lane 7, band 2) with parent vector clones migrating similar to f8-5 phage particles and f8-4/8mer (Figure 3, lane 8, band 3) with parent vector clones migrating similar to fd-cat phage particles (Figure 3, lane 3, band 1). No discrete bands corresponding to the size of the parent vector-containing phage particles were observed in the f8/9 library. To confirm that phage migration was dependent upon the exposed residues present on the p8, we tested the mobility of three landscape phage clones all containing the same sized genome but displaying different surface charged residues. As expected, the phage particles all migrated with reduced relative mobility (Figure 3, lanes 10, 11 and 12) as compared with the f8-5 phage particles (Figure 3, lane 2). These data demonstrate that modification of every copy of the p8 protein creates vastly different phage particles with unique physicochemical properties that affects their migration through a structurally homogeneous microenvironment with highly controlled properties.

### 3.2. Tissue Distribution of Landscape Phage Display Library

On the basis of our results of landscape phage migration through an agarose gel in response to an externally applied electric field, we reasoned that landscape phages would exhibit different pharmacokinetic and tissue-selective distribution profiles based on the unique physicochemical landscapes produced by each clone as compared with p3-type libraries displaying a single landscape composed of the wildtype p8 protein. Here, we studied the tissue distribution of two intravenously administered single doses of the f8/9 phage library in athymic (NCr-nu/nu) mice, with or without the human tumor xenograft of MDA-MB-231 cells, for 1 or 24 hours. Triple negative breast cancers correspond to ~10% to 20% of all breast cancer diagnoses and often exhibit a more aggressive phenotype with increased recurrence rates and worse survival as compared with other breast cancer subtypes [57]. We used MDA-MB-231 breast cancer cells in this study as a representative model of a basal-like, triple negative breast cancer subtype, negative for estrogen receptor (ER), progesterone receptor (PR), human epidural growth factor receptor 2 (HER2), and E-cadherin expression [58,59,60]. The nude mouse model used throughout the study are deficient in T cells, demonstrate a normal repertoire of functionally diverse B cells, but have elevated macrophage and NK cell populations [61]. These mice support the growth of tissue and tumor xenografts, including the MDA-MB-321 human breast cancer cell line used in this study, have been used previously to study the tissue distribution of p3-type phage libraries [44], and are commonly used strains in many laboratories for efficacy testing of new chemotherapeutic agents.

We first sought to determine the physiologically tolerated dosage of library to administer that would allow recovery of tissue-selective phage particles from all tissues while simultaneously reducing any dose-limiting toxicities of the library. Dilutions of the phage library, composed of ~4.0 × 10^10^ virions (high dose) or ~4.0 × 10^9^ virions (medium dose), were administered in a constant 0.2 mL of total volume to both groups of mice and observed for 1 hour or 24 hours before sacrifice. Twenty-four hours post-administration, no mortalities were observed in mice across either group. A slight decrease in body weight was observed in mice receiving the highest dose of library but was expected to be a nonlethal, transient change. No other gross clinical observations were observed. Overall, the f8/9 phage library was well tolerated and demonstrated no gross signs of acute toxicity over the study time period as was demonstrated with other types of phage libraries [2,18,62].

Next, we sought to quantify the distribution of phages in each tissue and compare the total number of infectious phage particles obtained by traditional titering in a suitable bacterial host to the total number of phage particles as determined using a phage-specific qPCR. We designed p8-specific primers which would produce a 160 bp DNA fragment after PCR amplification for any clone from a landscape phage display library and also span the degenerate insert region to subsequently allow for sequencing by Sanger or NGS techniques. As xenografts were expected to contain a mixture of human gDNA and mouse gDNA, we designed a qPCR with specificity towards ultra-conserved regions of the genome to normalize each sample to the number of mammalian genomes in each sample [63]. To validate this approach, we imposed several primer design constraints including: (1) spanning an exon-intron junction; (2) the conserved genomic region must be homologous across several species [human, mouse, canine, and cat]; (3) there should be no significant variation in chromosomal copy number for common breast cancer cell line models; and (4) selected primers must not amplify bacteriophage or bacterial gDNA. Here, we used the ultra-conserved region identified as UC.33, a 312 bp region within the human 1p21.3 chromosome band that contains 34 bases of exon 10 from an alternatively spiced variant of the PTBP2 gene and 278 bases of intergenic region [64]. This region of the chromosome (1p21) showed no significant changes in copy number variation in either MDA-MB-231 or MCF-7 cell profiles [65] and contained 100% sequence identity in all species tested. All primer sets designed in this study demonstrated specific and selective amplification of the indicated target, as confirmed by Sanger sequencing and generated reproducible efficiencies near 100% covering a broad range of dilutions.

**Figure 4:**
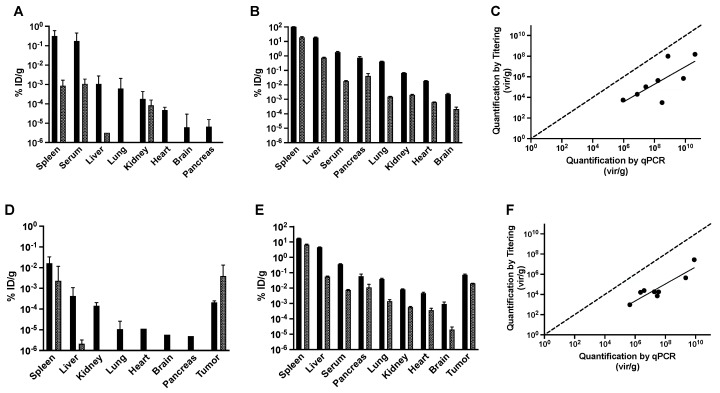
Quantification of phages in mouse tissues. Phage concentrations, represented as the percentage of injected dose recovered per gram of tissue (%ID/g), from isolated tissues after administration of a high dose of f8/9 library in (**A**–**C**) non-tumor bearing NCR-nu/nu mice as compared with (**D**–**F**) NCR-nu/nu mice bearing an MDA-MB-231 breast cancer xenograft. Biological titering of phages in *E. coli* cells (**A**,**D**) or physical quantification of phages by qPCR (**B**,**E**) after 1 hour (black, solid bars) or 24 hours (grey, hashed bars) of circulation. Bar charts display the mean ± standard deviation of technical replicates for each tissue [*N* ≈ 3] from a single representative mouse. Mean phage concentrations (vir/g) from matched tissue samples were plotted (**C**,**F**) and a linear regression model was fit for tissues recovered from an individual mouse after 1 hour (black).

After 1 hour of circulation, a large percentage of the injected phages in non-tumor bearing mice was found remaining in serum or accumulated within the spleen or liver, irrespective of dose. A smaller, but significant portion of the infective phages were also found within the lungs, kidney, and heart (Figure 4A and Figure S1). The tissue demonstrating the least recovery of phages after 1 hour using either dose was the brain. In general, when comparing matched tissue concentrations obtained by titering versus qPCR, trends were similar across all tissues, except from pancreas, and fit a linear regression model with high correlation (Figure 4C,F). We found a noticeable difference in quantification method for phages recovered from the pancreas after 1 hour. Phages recovered after 1 hour by titering were comparable to the concentrations recovered in the brain, whereas phages recovered by qPCR were higher, comparable to concentrations found in the serum or lungs. This would be expected as increased exposure time of phages to immune cells in vivo or similarly harsh conditions within the local tissue microenvironment may cause inactivation of p3, leading to the production of non-infectious particles and the inability to quantify by titering. This is demonstrated by the greater recovery of phage particles across all tissues by qPCR than titering (Figure 4C,F), as previously described in the literature [66]. Similar trends were observed after 24 hours with significantly fewer tissues containing any infectious phages, including lung, heart, brain, and pancreas. Titerable concentrations of phage particles were only found in spleen, serum, liver, and kidneys (Figure 4A). However, phage gDNA was still recovered in all tissues after 24 hours, suggesting that phages remain distributed within the tissues but rapidly lose their infectivity (Figure 4B). Similar patterns of tissue distribution and loss of phage infectivity were also observed following administration of the medium library dose after 24 hours (Figure S1A).

Similarly, in tumor-bearing mice, a large percentage of infectious phage particles were recovered in the spleen and liver after 1 hour of circulation, irrespective of dose, as seen in non-tumor bearing mice (Figure 4D). A smaller portion was found in the tumor, kidney, and lungs with a few infectious phages recovered from the heart, brain, and pancreas. We saw that quantification by qPCR produced the same relative distribution of phages throughout the tissues in tumor-bearing mice as compared with non-tumor bearing mice (Figure 4E), with a significant portion recovered in the tumor tissues. Again, many phages in the tissues were non-infectious after 24 hours and could only be recovered and quantified by qPCR.

### 3.3. Molecular Evolution of Tissue-Selective Peptides Displayed on Landscape Phages

To identify peptides that were selectively enriched within each tissue, we used targeted amplicon sequencing of p8-amplicon libraries generated from each tissue after quantification of phage genomes. Individual, tissue-specific p8-amplicon libraries were uniquely barcoded with multiplex adapters and pooled into a single sequencing reaction containing 10 different tissue samples. An average of ~1.5 million sequence reads (±~380,000 reads) per tissue library were generated following a single-end NGS reaction, demultiplexing, and parsing of tissue-specific libraries into a raw FASTQ file per tissue. We then analyzed each tissue-specific amplicon library using our p8-targeted bioinformatics workflow presented in Figure 2. Given the short length of our p8 amplicon, we were able to double the number of usable reads by identifying and processing both sense and anti-sense sequence reads following a single-end NGS reaction. Following removal of conserved p8-upstream and -downstream sequences from either the sense strand or anti-sense strand reads, the reverse complement of each anti-sense strand read was determined and added to the tissue-specific collection of sense reads. We found that ~80% of all reads passed initial filters for p8 sequence identity while also containing a recombinant nucleotide fusion of 27 bases correctly located for a clone derived from the f8/9 phage library. A 20% increase of correctly processed anti-sense reads, as compared with sense reads, was observed across all samples in multiple experiments. This suggests that processing of both sense and anti-sense reads is required for maximal recovery of reads in our workflow. Given the degenerate composition of the recombinant insert and the higher error rates associated with NGS techniques, we chose to filter and exclude reads from further analysis containing a low-quality score (Q). We analyzed the effect of quality score filtering on each sample and found a Q > 34 as ideal, allowing a compromise between maximal recovery of sequence reads and low error rate. Filtering reads with Q > 34 produced on average ~38,000 unique nucleotide variants and coding for ~30,000 unique peptide variants per tissue library.

**Figure 5:**
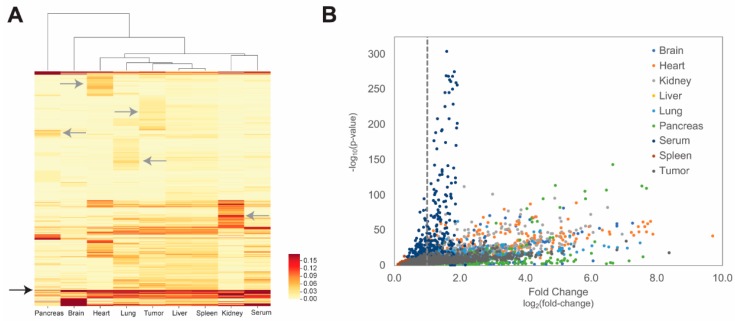
Identification of tissue-selective motifs by tissue. Tissue-selective motifs were visualized via (**A**) hierarchically clustered heatmap of motif occurrence within each tissue and (**B**) one-sided volcano plot visualizing motifs that were significantly increased in copy number as compared with the unselected f8/9 phage library within each tissue. (**A**) Tissue-normalized motif counts, expressed as a percentage of each recovered population from each tissue, were generated for each motif, and clustered based on similar motif frequency patterns across the tissues studied. Motif percentages were visualized in a heatmap with yellow bands representing low frequency motifs and red bands representing high frequency motifs. Nonspecific tissue motif clusters are indicated by black arrows and tissue-specific motif clusters are indicated by grey arrows. (**B**) Tissue-selective motifs were identified by plotting the log_2_(fold change) of tissue-specific motif occurrence as compared with library-normalized motif frequencies against the log_2_(p-value) as calculated by a Fisher’s exact test. A threshold of *p* ≤ 0.05 and 2-fold changes in motif occurrence (represented by the dashed, grey vertical line) were used to identify significantly accumulated motifs within each tissue.

We hypothesized that following in vivo evolution, trimer motifs located within each peptide would represent the shortest linear, non-gapped EBU that provides sufficient structure to support development of protein–protein interactions like SLiMs [67,68] and could be initially enriched for binding or navigation within the tissue microenvironment of each organ [18,69]. To reveal 3-mer motifs that were enriched in each tissue, we calculated the occurrence frequency of each motif within each studied tissue. Motif frequencies were visualized using a hierarchically clustered heatmap (Figure 5A), in which each motif is represented as a single row and the frequency within each tissue is indicated by the darkness of the band. Each row was clustered by similar frequencies of motif occurrence between tissues. Several nonspecific clusters of motifs with relatively high occurrences were found in all organs, as indicated by the black arrow. We also identified several clusters of motifs enriched in a tissue-specific manner, as indicated by the grey arrows, suggesting interaction of these tissue-selective binding motifs with proteins expressed in a tissue-specific manner or similar functional properties of the local tissue microenvironment. In several tissues, there were highly differentiated clusters of motifs that could be visualized, as in the kidney, heart, and tumor, suggesting unique structural components of the microenvironment that play a role in specifically enriching these motifs. We observed that the distribution of motifs from the f8/9 phage library is most similar within the liver and spleen suggesting their similar role in providing clearance mechanisms of phages from systemic circulation or highly structural similarities in tissue extracellular matrix (ECM) protein networks [70]. Given the lack of tissue-specific motif enrichment, we speculate that most phages in the liver and spleen were accumulated via motif-independent processes that were largely independent upon the peptide structure displayed on p8. From these data, we demonstrate that 3-mer motifs were enriched in a tissue-specific manner at the earliest stages of in vivo evolution, as confirmed by previous studies [33,52,69].

Next, we sought to identify tissue-selective motifs that were significantly enriched in each tissue as compared with their occurrence in the unselected, primary f8/9 phage library. Selectivity of each motif was calculated as suggested previously [52], with the frequency of each motif compared to the frequency in a normalizing sample using a 2 × 2 contingency table to calculate a one-sided Fisher’s exact test for each motif. Motif fold-change was calculated for each motif comparing the frequency obtained in the tissue as compared with the frequency in a normalizing sample. We reasoned that normalization by the unselected library sample would give the greatest diversity of viable motif structures as compared with motifs identified in phages from all other tissues or serum. As demonstrated for identification of tumor-selective motifs in Figure S2, most of the motifs were captured using any normalization strategy and library normalization was used for all other figures. Identified motifs were plotted in a one-sided volcano plot to visualize tissue-selective motifs with greater abundance and high statistical significance (Figure 5B). Several tissue-selective motifs were enriched as compared with the corresponding frequency observed in either the unselected f8/9 library or in all other tissues. On average, we found approximately 480 significantly enriched 3-mer motifs per tissue with varying degrees of motif occurrence. As there was no amplification of phages through bacteria before NGS, we expect no bias of motif frequencies based on fast growing phage particles except those generated during the initial library construction. On the basis of these data and the proposed algorithm, we demonstrate that tissue-selective motifs can be identified using NGS as early as the first round of selection in vivo after 1 hour of circulation.

### 3.4. Molecular Evolution of Tumor-Selective Peptides Displayed on Landscape Phages

**Figure 6:**
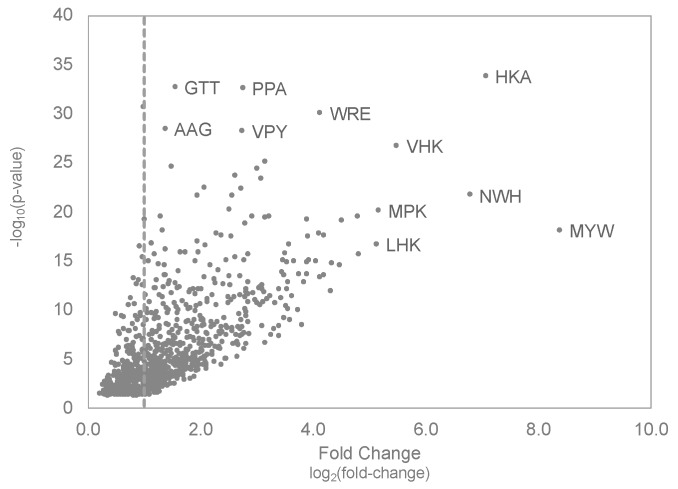
Identification of tumor-selective motifs. MDA-MB-231 breast cancer cell-selective motifs were identified by comparing relative motif frequencies recovered in the tumor compared motifs recovered from the unselected f8/9 phage library. Here, the tissue-specific fold change was plotted against the *p*-value obtained from a Fisher’s exact test comparing the frequency differences between the two motif populations.

After identifying tissue-selective 3-mer motifs, we hypothesized that several of these motifs would be found enriched within the same peptide sequence, as would be expected in our model of combinatorial avidity selection [33]. Using the significantly enriched motifs identified in the tumor tissue, we studied the interaction of tumor-selective motifs with each other to identify important peptide structures that may be used for selective migration and navigation into the tissue microenvironment. From the population of 1005 significantly enriched tumor-selective motifs, 576 motifs showed a greater than two-fold increase in relative occurrence, as summarized in (Figure 6). We observed some motifs, like “MYW” or “WRE”, that were significantly enriched in the tumor-selective population of phages but did not appear to share any connections with neighboring motifs based on their occurrence. However, some motifs, like “HKA” and “VHK”, demonstrated similar increases in occurrence and significance suggesting selected peptides may contain both motifs forming a larger “VHKA” motif. Analysis of the peptide structures recovered from phages accumulating in the tumor revealed that 27 peptides contained both EBUs and formed the larger “VHKA” motif.

**Figure 7:**
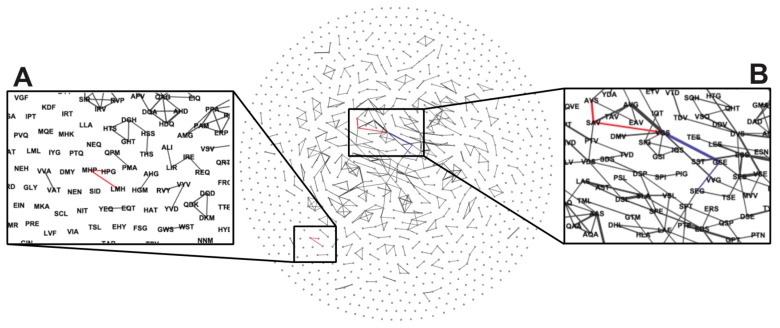
Interaction networks of tumor-selective EBU. Tumor-selective EBUs were plotted as a network graph as nodes that were connected by weighted edges (thicker line between nodes) representing the number of peptides containing both EBUs within the same structure. (**A**) Simple EBU network, noted by the highlighted red path, demonstrating linkage of individual motifs with limited diversity of neighboring EBU interactions. (**B**) Complex EBU network, noted by the two highlighted paths (red and blue), demonstrating linkage of motifs with diverging paths with neighboring EBUs.

We performed network analysis to study the complete set of interactions for each significantly enriched EBU within each phage peptide sequence recovered from the tumor and represented tumor-selective EBUs as points (or nodes) within the network. The EBUs found within the same peptide structure were linked by a weighted edge representing the number of unique times the two EBUs were structurally connected in the same peptide. From the resulting network graph (Figure 7), we observed the following three types of interactions: (a) isolated EBUs, (b) simple EBU networks, and (c) complex EBU networks. Isolated EBUs, as demonstrated by the “MYW” motif above, represent EBUs that were enriched but did not demonstrate preference for specific interactions with other significantly enriched EBUs. Analysis of peptide structures containing the “MYW” EBU (Table 1) demonstrates that there is strong positional preference for the EBU that may be relevant for functional activity. Similarly, there are preferred neighboring amino acid residues surrounding the EBU, but variation is tolerated. Simple EBU networks, as demonstrated by the “LMH/MHP/HPG” motifs (Figure 7A), represent interactions between EBUs with a strong preference to enrich specific neighboring amino acids and often form longer motifs by extension of a primary EBU. These simple EBU networks most closely resemble enrichment of a functional peptide by affinity maturation (Table 1).

Complex EBU networks, as demonstrated by the interactions of the “VGS” motif (Figure 7B), represent enrichment of multiple significant EBU interactions within a population of peptides. Here, two divergent peptide populations are demonstrated, peptides containing “VGS/AVS” motifs and those containing “VGS/SEG” motifs (Table 1). The combination of the “VGS/AVS” motifs in these peptides appears to follow enrichment via affinity maturation but demonstrates that this functional domain may not have a strong preference for position within the displayed peptide. Alternatively, the combination of “VGS/SEG” motifs is much more variable suggesting enrichment of a functional peptide domain by the combination of two independent EBUs. The EBUs in this family of peptide structures do not have a strong preference for position within the peptide, nor do they have a strong preference for linkage distance between the two EBUs. Here, the “VGS” EBU is found more N-terminally than the “SEG” EBU but can be separated with a gap of two amino acids or found as two consecutive EBUs. (Table 1).

## 4. Discussion and Conclusions

In this study, we considered the following two major factors that could influence the evolution of the f8/9 landscape phage library in mice: (1) its multidisperse characteristics as revealed in soft agar electrophoresis and (2) organ- and tumor-specific targets for phage binding interactions that can arise from organ- or tissue-specific gene expression. We showed that modified p8 proteins displaying a fusion peptide along the length of the filamentous phage particle would create vastly different particles, each containing unique physicochemical features and supporting complex interactions within a defined microenvironment. As reflected in our in vitro migration study, the presence of random fusion peptides with charged amino acids in landscape phages results in significant changes in the ionization state of the phage particle leading to differences in surface charge and their subsequent ability to migrate through an agarose gel, demonstrated previously with isolated clones [41]. This unique, emergent feature of landscape phages cannot be translated to p3-type libraries as the occurrence of p3 in only five copies limits the ability of a displayed fusion peptide to significantly change the physicochemical properties of the entire particle. It can be envisioned that local changes in pH within a tissue microenvironment may modify the surface charge of landscape phage particles, exposing a previously hidden binding epitope or also hiding a previously accessible binding epitope allowing enhanced penetration or retention within a particular microenvironment [71,72].

Following an extensive meta-analysis study of nanoparticle biocompatibility in vivo, it was concluded that functional nanocarriers had a slightly negative surface charge, moderate to high solubility, and particle sizes in the range of ~100–200 nm in diameter [73]. Given that landscape phage libraries display peptide fusions that can dramatically alter the surface profile of the phage, we hypothesized that libraries of phage particles represent ideal nanoparticle systems to test the effects of a displayed peptide on tissue-selective accumulation within the local microenvironment while maintaining other critical parameters like particle size and shape. In our study, we observed similar trends of tissue accumulation as reported previously for p3-type libraries [44]. Analysis of 3-mer frequency in each tissue revealed that a significant portion of motifs were shared across all tissues, leading to nonspecific uptake and clearance or had very low occurrences. We were able to identify several families of motifs that were enriched in a tissue-selective manner. Some of the identified motifs corresponded with previously identified tissue-selective motifs [52]. However, we identified many motifs that were not reported, suggesting that the type of peptide display will have a significant effect on tissue accumulation or pharmacokinetics.

The underlying hypothesis in our in vivo evolution study of landscape phages is based on the assumption that tissue-specific gene expression provides a context-dependent pathway for development of tissue specificity [74,75,76]. These tissue-specific gene expression profiles provide transcriptional and translational control for tissue-specific synthesis of corresponding proteins and their underlying protein-domain networks [77]. It was suggested that components of these protein-domain networks could be identified through in vitro or in vivo targeted evolution of EBU/SLiM-harboring phage displayed peptide libraries such as the multivalent landscape phage library used in this project [31,33]. In accordance with this hypothesis, we identified the following three major types of EBU motifs: (1) motifs common for all tissues; (2) motifs specific for organs with functionally related tissues and similar correlated patterns of co-expressed genes; and (3) motifs unique to single tissues with unique functions, which may be related to tissue-specific gene expression profiles within the tissues or to specific physical features of the local microenvironment. Here, we demonstrated that the frequencies of several 3-mer motifs are shared across all tissue types (Figure 5A, black arrow) and can be representative of interactions with protein products of “housekeeping-like” genes expressed at constant levels across all tissue types, such as those involved in metabolism. Similar patterns of motif enrichment could be identified in tissues with functionally-related roles and gene expression patterns. This can be visualized by enrichment of similar motifs patterns between the liver and spleen with 25 significantly enriched motifs being shared between both tissues (Figure 5A). These organs are expected to contain cell types or structures within the local microenvironment that have common biological functions, representing a theoretical construct for targeting to a multi-organ system. Finally, we demonstrated motif enrichment within single tissues, including the tumor xenograft, which suggests there are tissue-specific genes that are overexpressed or structures in the microenvironment that allow for tissue-selective accumulation of phages. This is demonstrated by 3-mer motifs like “MYW” that are only enriched within the tumor microenvironment and suggested to play a role in selective binding of a tumor-specific marker.

Through this study, we demonstrated that landscape phage display libraries are a rich source of nanobioparticles with unique physicochemical properties bestowed on each phage. These unique libraries can be evolved for novel emergent features, including navigation to particular tissues or organs. Further study of directed evolution of landscape phage libraries in human patient-derived xenograft (PDX) mouse models, which mimic the tumor microenvironment of human patients more closely than isolated cell culture-based models, will result in the discovery of a variety of peptides, their EBU/SLiM fragments, and counterpart partner protein domains in a clinically-relevant system, which can be used to develop peptide chemotherapeutic leads or targeted delivery vehicles with improved activity [78,79].

## Figures and Tables

**Table 1:** Peptides identified by network analysis of EBUs (indicated in bold).

MYW	LMH/MHP/HPG	VGS/AVS	VGS/SEG
A**MYW**DRASD	D**LMHGP**VMD	**VGSAVS**NEH	AS**VGSEG**DL
D**MYW**DGASD	D**LMHPG**AAD	**VGSAVS**SEH	AS**VGSEG**ST
D**MYW**DKASD	D**LMHPG**AEG	VTD**VGSAVS**	DPSL**VGSEG**
D**MYW**DRADD	D**LMHPG**AID		DSSL**VGSEG**
D**MYW**DRALA	D**LMHPG**AKD		**VGSEG**MVID
D**MYW**DRALD	D**LMHPG**AMA		**VGSEG**STTL
D**MYW**DRAPD	D**LMHPG**AMD		**VGS**IQ**SEG**T
D**MYW**DRASA	D**LMHPG**AME		**VGS**TQ**SEG**T
D**MYW**DRASD	D**LMHPG**AMG		
D**MYW**DRASG	D**LMHPG**AMH		
D**MYW**DSASD	D**LMHPG**AMN		
D**MYW**DSPSS	D**LMHPG**AMS		
D**MYW**GRASD	D**LMHPG**AND		
G**MYW**DRASD	D**LMHPG**ASE		
V**MYW**DRASD	D**LMHPG**ATD		
Y**MYW**DRASD	G**LMHPG**AMD		

## Supplementary Materials

The following are available online at https://www.mdpi.com/1999-4915/11/11/988/s1, Figure S1: Quantification of phages in mouse tissues (medium dose); Figure S2: Comparison of tissue-selective motif normalization strategy; Table S1: Filamentous phage library summary.
