# Supplementary material for: Evolution of a Landscape Phage Library in a Mouse Xenograft Model of Human Breast Cancer
Source: Viruses. 2019 Oct 26;11(11):988. doi: 10.3390/v11110988 (PMC6893515; doi:10.3390/v11110988)
Supplement: Supplementary file 1 [file viruses-11-00988-s001.pdf]

Supplemental Materials

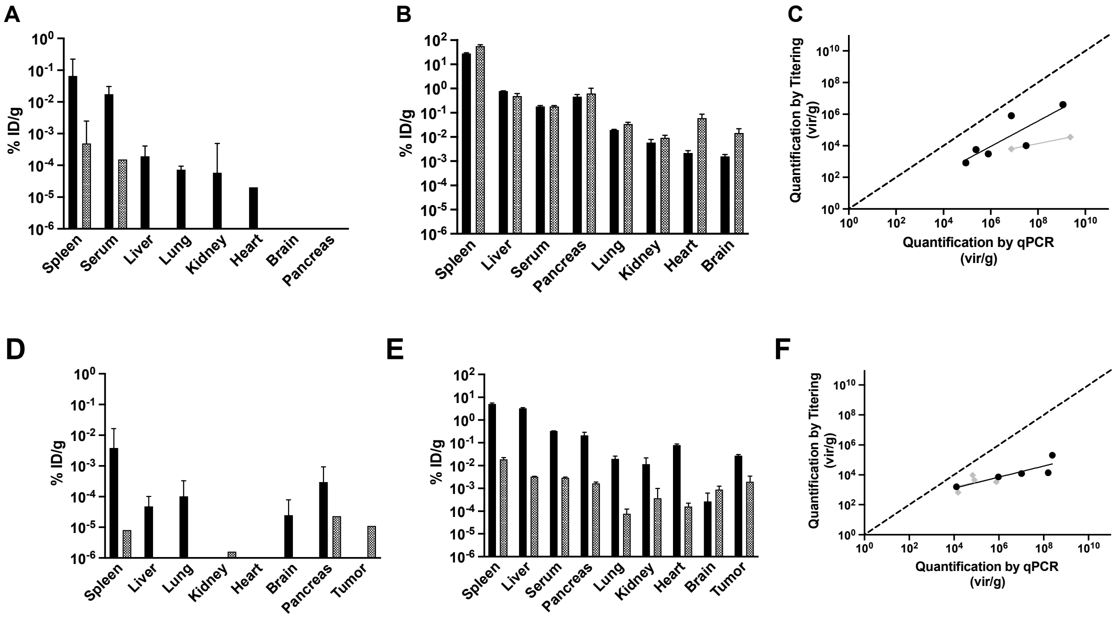

Figure S1: **Quantification of Phages in Mouse Tissues (Medium Dose).** Phage concentrations, represented as the percentage of injected dose recovered per gram of tissue (%ID/g), from isolated tissues recovered after administration of a medium dose of f8/9 library in (**A-C**) non-tumor bearing NCR-*nu*/*nu* mice compared to (**D-F**) NCR-*nu*/*nu* mice bearing an MDA-MB-231 breast cancer xenograft. Biological titering of phages in *E. coli* cells (**A & D**) or physical quantification of phages by qPCR (**B & E**) after 1 hour (black, solid bars) or 24 hours (grey, hashed bars) of circulation. Bar charts display the mean ± standard deviation of technical replicates for each tissue [N≈3] from a single representative mouse. Mean phage concentrations (vir/g) from matched tissue samples were plotted (**C & F**) and a linear regression model was fit for tissues recovered from an individual mouse comparing 1 hour (black) and 24 hours (grey).

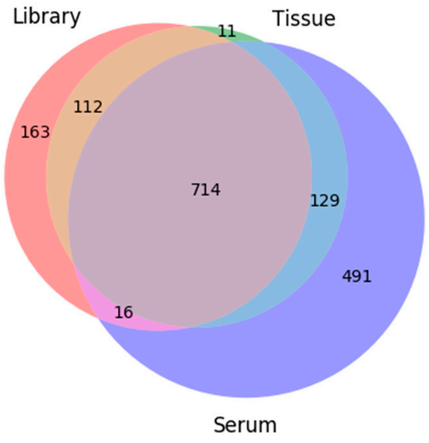

Figure S2: Comparison of Tissue-Selective Motif Normalization Strategy. Venn diagram comparing the number of significantly enriched tumor-selective motifs based on a) unselected f8/9 phage library, b) other recovered tissues, or c) serum.

Table S1: Filamentous Phage Library Summary

Sample	Genome Size (bp)	Isoelectric Point (Exposed p8)
**Vectors**		
fd-tet Phage [1]	9,183	3.69
f8-5 Phage [2]	9,183	3.58
C8-4 Phage	7,775	3.69
**p3-type**		
f3-6mer Phage Library [3]	9,225	3.69
f3-15mer Phage Library [4]	9,252	3.69
**p88-type**		
f88-15mer Phage Library [5]	9,273	Mixture
**p8-type**		
f8-5/6mer Phage Library [2]	9,183	Mixture
C8-4/8mer Phage Library	7,790	Mixture
f8/9 Phage Library [6]	9,198	Mixture
DMPGTVLP Phage [7]	9,198	3.64
EPSQSWSM Phage [8]	9,198	3.78
VPEGAFSS Phage [9]	9,198	3.78

Reference

1. Zacher, A.N., 3rd; Stock, C.A.; Golden, J.W., 2nd; Smith, G.P. A new filamentous phage cloning vector: fd-tet. *Gene* **1980**, *9*, 127-140.
2. Petrenko, V.A.; Smith, G.P.; Mazooji, M.M.; Quinn, T. Alpha-helically constrained phage display library. *Protein Eng* **2002**, *15*, 943-950.
3. Scott, J.K.; Smith, G.P. Searching for peptide ligands with an epitope library. *Science* **1990**, *249*, 386-390, doi:10.1126/science.1696028.
4. Nishi, T.; Budde, R.J.; McMurray, J.S.; Obeyesekere, N.U.; Safdar, N.; Levin, V.A.; Saya, H. Tight-binding inhibitory sequences against pp60(c-src) identified using a random 15-amino-acid peptide library. *FEBS Lett* **1996**, *399*, 237-240, doi:10.1016/s0014-5793(96)01329-4.
5. Choukri, S.; Smith, G.P. Construction of f88-15mer Phage Library (Genbank AF246448). 2000.
6. Kuzmicheva, G.A.; Jayanna, P.K.; Sorokulova, I.B.; Petrenko, V.A. Diversity and censoring of landscape phage libraries. *Protein Eng Des Sel* **2009**, *22*, 9-18, doi:10.1093/protein/gzn060.
7. Fagbohun, O.A.; Bedi, D.; Grabchenko, N.I.; Deinnocentes, P.A.; Bird, R.C.; Petrenko, V.A. Landscape phages and their fusion proteins targeted to breast cancer cells. *Protein Eng Des Sel* **2012**, *25*, 271-283, doi:10.1093/protein/gzs013.
8. Bedi, D.; Gillespie, J.W.; Petrenko, V.A. Selection of pancreatic cancer cell-binding landscape phages and their use in development of anticancer nanomedicines. *Protein Engineering Design & Selection* **2014**, *27*, 235-243, doi:10.1093/protein/gzu020.
9. Petrenko, V.A.; Smith, G.P. Phages from landscape libraries as substitute antibodies. *Protein Eng* **2000**, *13*, 589-592, doi:10.1093/protein/13.8.589.
